# Health-related quality of life and behavior-related lifestyle changes due to the COVID-19 home confinement: Dataset from a Moroccan sample

**DOI:** 10.1016/j.dib.2020.106239

**Published:** 2020-08-27

**Authors:** Asmaa Azizi, Doha Achak, Khalid Aboudi, Elmadani Saad, Chakib Nejjari, Youness Nouira, Abderraouf Hilali, Ibtissam Youlyouz-Marfak, Abdelghafour Marfak

**Affiliations:** a Laboratory of Health Sciences and Technology, Higher Institute of Health Sciences, Hassan First University of Settat, Morocco; b Epidemiology, Clinical Research and Community Health, Faculty of Medicine and Pharmacy of Fez, University Sidi Mohammed Ben Abdellah, Fez, Morocco; c International School of Public Health, Mohammed VI University of Health Sciences (UM6SS), Casablanca, Morocco; d Higher Institute of Nursing Professions and Health Technology of Rabat, Morocco

**Keywords:** COVID-19 pandemic, Health-related quality of life, EQ-5D-5L, Behavior-related lifestyle, Home confinement, ARI, Absolute Risk Increase, ARR, Absolute Risk Reduction, HRQoL, Health-Related Quality of Life

## Abstract

The Severe Acute Respiratory Syndrome Coronavirus 2 (SARS-CoV-2) virus initially appeared in Wuhan, Hubei Province, China has caused a novel coronavirus disease (COVID-19) [1]. The disease is rapidly spread around the world causing thousands of deaths and posing critical challenges for public health and clinical research in the world. The outbreak was declared by the World Health Organization (WHO) as a public health emergency of international concern. Nowadays, there are more than 18 millions of confirmed cases of coronavirus across the world with a total of 702,903 deaths [2]. In Morocco, there are 28,500 confirmed cases and 345 deaths. Forecasts for the cumulative number of confirmed, recovered, active and death cases were recently provided [3]. To manage the pandemic spread several countries adopted proactive and preventive measures including home confinement of the population. However, there is evidence that these measures, particularly home confinement, can cause unprecedented disruption in the well-being of the population. Being forced to stay at home and the daily activities’ restrictions could impact the citizens’ health-related quality of life (HRQoL) and behavior-related lifestyle. It has been reported that the COVID-19 pandemic has an impact on psychological behaviors [4], mental health [5] and anxiety/depression [6]. The Moroccan population was under home confinement from March 20, 2020. Assessing rapidly and simply the HRQoL during crisis such as the home confinement is a challenge of interest to provide speedy information to authorities which allow best management of damages yielding in crisis situation. The EQ-5D instrument is a generic questionnaire developed by the Euroqol group for measuring the HRQoL by combining five health dimensions (mobility, self-care, usual activities, pain/discomfort, and anxiety/depression) [7]. The EQ-5D instrument is translated into more than 300 languages and exhibit good reliability and validity in both patients and general population. Herein, we used the EQ-5D-5L instrument to assess for the first time the impact of the home confinement on the HRQoL. Also, we evaluated the changes in behaviors by asking some questions related to lifestyle before and during the home confinement. These data can help the Moroccan authorities and other countries to more understand the impact of this crisis on citizens and therefore to set up adequate protocols for managing the post-confinement or possible future crisis. We provided two datasets: (1) data we collected before confinement from a sample of 484 individuals describing their HRQoL [8] and (2) data we collected during the home confinement period from a sample of 537 individuals describing their HRQoL and behavior-related lifestyle.

## Specifications Table

**Table:** 

Subject	Public health and health policy
Specific subject area	Health-related quality of life and behavior-related lifestyle
Type of data	Table Graph
How data were acquired	The data were collected using an online survey (Supplementary Material, Table 1S)
Data format	Data are in raw format and provided in an Excel file
Parameters for data collection	Data were collected considering: (1) the health-related quality of life using the EQ-5D-5L questionnaire and (2) behavior-related lifestyle represented by 24 questions. Two circumstantial conditions were considered (before and during the home confinement).
Description of data collection	Two datasets were provided: (1) EQ-5D-5L data from 484 individuals before confinement [8] and (2) EQ-5D-5L and behavior-related lifestyle from 573 individuals during the home confinement. The latter dataset was collected using an anonymous online questionnaire in Moroccan Arabic dialect between May 9, and May 30, 2020. The questionnaire was designed following Helsinki's Declaration of ethics. On the main page, the purpose of the data collection and a letter of consent were presented. Access to the questionnaire was only given if the respondent consented to participate.
Data source location	Institution: Laboratory of Health Sciences and Technology, Higher Institute of Health Sciences, Hassan First University of Settat City: Settat Country: Morocco
Data accessibility	All data were provided with the article in supplementary Excel file

## Value of the Data

• These data provide information on the health-related quality of life and behavior-related lifestyle, which is important for understanding the home confinement impact due to COVID-19 on the health population.
• The authorities can benefit from these data to set up adequate protocols for the post-confinement and a possible future similar crisis.
• Other researchers around the world can used these data to conduct comparison in other countries.
• These data provide may complete the knowledge on the impact of the COVID-19 outbreak and therefore more managing this epidemic crisis.

## 1 Data Description

### 1.1 Soci*o-demographic characteristics*

A total of 537 participants (338 females and 199 males) completed the online questionnaire during the home confinement (Table 1). The mean age of the participants was 33.19 ± 12.14 years. Half of them were single (50.3%), 44.1% married, 4.8% divorced and 0.7% widowed. The majority of participants (57.7%) don't have children, while 33.1% have less than four and 9.1% have more than four children. With respect to the education level, 85.8% have a high education level compared to 0.9% who has never gone to school. The distribution of participants according to the profession revealed that 63.3% are working, 19.6% are students and 17.1% with no occupation. About three quarter of the participants (78.6%) had a medium socio-economic level. Twenty percent of participants are under medical treatment for diabetes, cardiovascular disease, kidney disease, anemia and allergy.

**Table 1:** Socio demographic characteristics of the participants during the home confinement (n=537).

**Variables**		**n (%)**
**Sexe**		
	Female	338 (62.9)
	Male	199 (37.1)
**Age**		
	18-30	286 (53.3)
	31-50	187 (34.8)
	>50	64 (11.9)
**Marital status**		
	Single	270 (50.3)
	Married	237 (44.1)
	Separated	26 (4.8)
	Widowed	4 (0.7)
**Educational status**		
	Illiterate	5 (0.9)
	Primary education	14 (2.6)
	Secondary education	57 (10.6)
	University education	461 (85.8)
**Profession**		
	Student	105 (19.6)
	worker	340 (63.3)
	No occupation	92 (17.1)
**Socio economic level**		
	Low	88 (16.4)
	Medium	422 (78.6)
	High	27 (5.0)
**Number of children**		
	0	310 (57.7)
	≤ 4	178 (33.1)
	>4	49 (9.1)
**Presence of disease**		
	Yes	110 (20.5)
	No	427 (79.5)

### 1.2 Health-related quality of life during versus before the home confinement

**Fig. 1:**
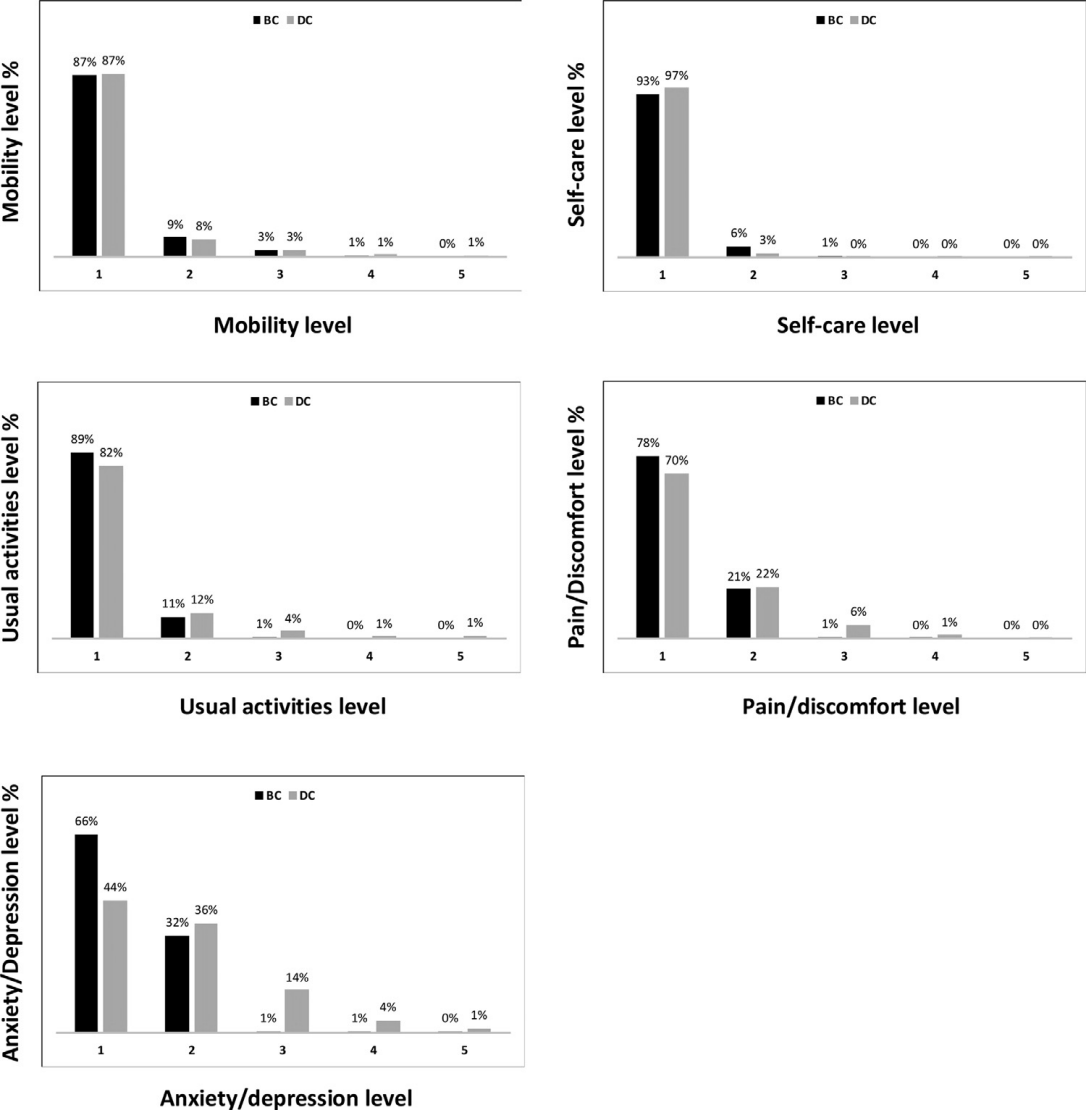
Profile of health-related quality of life among individuals before confinement (n = 484) and those during the home confinement (n = 537). BC: before confinement, DC: during confinement.

To evaluate the impact of the home confinement on the HRQoL, the data collected from participants during confinement were compared to a dataset of 484 participants from another study we carried out before confinement using the EQ-5D-5L instrument [8]. The EQ-5D-5L responses’ distributions were presented on Fig. 1. We observed that during confinement (before confinement) the percentages of don't have problems in the five health dimensions were 87% (87%), 97% (93%), 82% (89%), 70% (78%) and 44% (66%) for mobility, self-care, usual activities, pain/discomfort and anxiety/depression, respectively.

The comparison between the two samples showed that participants during confinement had lower scores of HRQoL on both utility (0.86; *P<0.0001*) and VAS (80.33; *P<0.0001*) compared to participants before confinement (utility=0.91 and VAS=88.75). In order to understand this difference, we compared each EQ-5D-5L dimension separately using the Improved RIDIT approach (Table 2). There was no significant impact of the home confinement on mobility and self-care. However, the home confinement increased by about ARI = 7.1% [2.7%; 11%] the problems in doing usual activities (odds_Ordinal_ = 1.74 [1.37; 2.23]). Also, during the confinement participants were observed to have odds_Ordinal_ = 1.56 times more pain/discomfort compared to individuals before the confinement. The anxiety/depression dimension was increased during the home confinement by 27.6% [21.2%; 33.9%] (odds_Ordinal_ = 2.75 [2.32; 3.27]).

**Table 2:** Improved RIDIT analysis of the EQ-5D-5L health-related quality of life dimensions of individuals during the home confinement versus individuals before.

	${\hat{\pi }}^{-}$	${\hat{\pi }}^{0}$	${\hat{\pi }}^{+}$	ARI[95% CI]	*P-*value	Odds ordinal[95% CI]
Mobility	0.119	0.765	0.117	-0.002 [-0.043; 0.039]	0.396	0.98 [0.67; 1.42]
Self Care	0.066	0.902	0.032	-0.034 [-0.061; 0.007]	0.067	0.48 [0.10; 2.42]
Usual Activities	0.094	0.741	0.165	0.071 [0.027; 0.114]	0.003	1.74 [1.37; 2.23]
Pain/Discomfort	0.158	0.595	0.247	0.089 [0.034; 0.143]	0.002	1.56 [1.26; 1.93]
Anxiety/Depression	0.157	0.410	0.433	0.276 [0.212; 0.339]	<0.0001	2.75 [2.32; 3.27]

CI: Confidence Interval and ARI: Absolute Risk Increase.

### 1.3 Association between socio-demographic characteristics and HRQoL during the home confinement

Determinants of HRQoL of participants during the home confinement due to the COVID-19 were illustrated in Table 3 and Table 2S (Supplementary Materiel). Females had lower scores of HRQoL on both utility (0.85; *P*=<0.0001) and VAS (78.49; *P*=0.004) compared to males (utility=0.89 and VAS=83.78). The comparison of the five EQ-5D-5L dimensions revealed that females had more mobility problems, pain/discomfort and anxiety/depression compared to males (Table 2S). In contrast, self-care and usual activities dimensions were not associated to gender. The data showed that there was no significant impact of age on the participants’ HRQoL. However, the marital status was significantly associated to EQ-5D-5L utility (*P*=0.002) and VAS (*P*=0.005) scores, the widowed participants had the worst HRQoL (utility=0.43 and VAS=48.75) compared to single (utility=0.87 and VAS=80.09), married (utility=0.86 and VAS=81.43), and separated (utility=0.89 and VAS=80.15) participants. In addition, mobility, self-care, usual activities and pain/discomfort dimensions were the most affected. In relation to education level the participants with university education had the higher EQ-5D-5L utility score (0.88; *P*<0.0001) compared to participants with secondary (0.80), primary (0.73) and without (0.67) education level. Participants with no occupation had lower EQ-5D-5L utility score (0.81) compared to students (0.88) and participants with professional activities (0.87). Concerning the number of children, there was a significant association between the number of children and the EQ-5D-5L utility score (*P*=0.003). Indeed, more the number of children increase, more the HRQoL was negatively affected. With respect to the socio-economic level, the statistical analysis revealed that there was no significant impact on both EQ-5D-5L utility (*P*=0.104) and VAS scores (*P*=0.404). Participants under treatment was negatively affected regarding the EQ-5D-5L utility (0.80; *P*=<0.0001) and VAS (74.47; *P*=0.002) scores compared to healthy participants (utility=0.88 and VAS=81.99). Mobility, autonomy, and pain/discomfort were the three dimensions most affected in sick participants (Table 2S).

**Table 3:** Association between health-related quality of life and the socio-demographic characteristics of individuals during the home confinement.

Variables	N (%)	EQ 5D_index_		EQ 5D_VAS_	
		Mean (SD)	*P*-value	Mean (SD)	*P*-value*
**Sexe**			**<0.0001**		**0.004**
Female	338 (62.9)	0.85 (0.19)		78.49 (19.66	
Male	199 (37.1)	0.89 (0.15)		83.78 (15.70)	
**Age**			0.860		0.965
18-30	286 (53.3	0.87 (0.17)		79.81 (19.75)	
31-50	187 (34.8)	0.87 (0.15		81.70 (15.96	
>50	64 (11.9)	0.83 (0.25)		79.64 (18.46	
**Marital status**			**0.002 (**a**)**		**0.026 (**a**)**
Single	270 (50.3)	0.87 (0.16)		80.09 (19.19)	
Married	237 (44.1)	0.86 (0.18)		81.43 (17.70)	
Separated	26 (4.8)	0.89 (0.13)		80.15 (13.87)	
Widowed	4 (0.7)	0.53 (0.43)		48.75 (14.36)	
**Educational status**			**<0.0001 (**b**)**		0.056
Illiterate	5 (0.9)	0.67 (0.45)		76.80 (28.44)	
Primary education	14 (2.6)	0.73 (0.28)		69.29 (24.72)	
Secondary education	57 (10.6)	0.80 (0.22)		77.49 (22.98)	
University education	461 (85.8)	0.88 (0.16)		81.20 (17.40)	
**Profession**			**0.006 (**c**)**		0.372
No occupation	92 (17.1)	0.81 (0.23)		78.02 (21.69)	
Student	105 (19.6%)	0.88 (0.16)		80.57 (19.15)	
Worker	340 (63.3%)	0.87 (0.16)		81.07 (17.26)	
Number of children			**0.003 (**d**)**		0.422
0	310 (57.7)	0.87 (0.16)		80.35 (18.98)	
1-4	178 (33.1)	0.87 (0.15)		81.42 (17.05)	
>4	49 (9.1)	0.78 (0.27)		77.53 (20.04)	
**Socio economic level**			0.104		0.404
Low	88 (16.4)	0.90 (0.11)		84.52 (18.42)	
Medium	422 (78.6)	0.85 (0.19)		79.03 (18.55)	
High	27 (5.0)	0.90 (0.10)		89.37 (11.99)	
**Presence of disease**			**<0.0001**		**0.002**
No	110 (20.5)	0.88 (0.16)		81.99 (17.20)	
Yes	427 (79.5)	0.80 (0.22)		74.47 (21.77)	

⁎ Kruskal-Wallis test

a Widowed

b University education

c No occupation and

d Number of children >4.

### 1.4 The behaviors’ changes during the home confinement

Table 4 summarizes some behaviors related to lifestyle of individuals before and during the home confinement. Regarding the eating habits, the number of meals was shifted from 3-4 per day before confinement to 1-2 and 5-6 per day during confinement (*P*=0.013). The percentage of snacking was increased from 31.3% before confinement to 39.5% during confinement (*P*=0.03). With respect to the quality of sleep, the usual bedtime was changed from 10:00 p.m-12:00 a.m. (61.5%) before the confinement to 12:00 a.m-2:00 a.m. (49.0%) and after 2:00 a.m. (29.2%) during confinement (*P*<0.0001). The usual wake-up time, before confinement was at 6:00 a.m. to 8:00 a.m. (57.9%); however, during confinement the wake-up time was moved to 8:00 a.m-10:00 a.m. (41.9%), and 10:00 a.m-12:00 p.m. (29.1%) (*P*<0.0001). The length of time spent napping during confinement was increased twice for the interval of 1-2 hours and five times for more than 2 hours compared to before confinement (*P*<0.0001). The time spent to physical activity was decreased during the home confinement. Also, the individuals were observed to practice less physical activity during the confinement period (*P*<0.0001). However, participants spent more time to their daily hygiene household activities during confinement (*P*=0.009). The E-working percentage was shifted from 15.8% before the home confinement to 51.4%. Also, the daily time spent to E-working was increased during confinement for the interval of 2-4h (17.9% vs 5.6% before) and 4-6h (11.7% vs 5.2% before). The participants were observed to spent more time per day tracking information and using their smart phones during the confinement (*P*<0.0001). The participants reported during the home confinement they spent more time in daily activities with their family members (*P*<0.0001). Twenty four percent of individuals reported they were not satisfied with their life during the home confinement, while there was only 7.1% did not satisfied before the confinement. The missed activities to the majority of individuals during the confinement situation were: go to the sport club and leisure areas, go to the mosque, visiting family and friends, shopping and go to restaurants (Table 3S).

**Table 4:** Comparison between the individuals’ behaviors before and during the home confinement.

	Before confinementn (%)	During confinementn (%)	P-value*
**Meal per day**			
1-2	74 (13.8)	131 (24.4)	**0.013**
3-4	448 (83.4)	367 (68.3)	
>=5	15 (2.8)	39 (7.3)	
**Snacking per day**			
None	168 (31.3)	212 (39.5)	**0.030**
1-2	294 (54.7)	242 (45.1)	
3-4	60 (11.2)	74 (13.8)	
>=5	15 (2.8)	9 (1.7)	
**Interval between Meals**			
1-2	63 (11 .7)	69 (12.8)	0.183
3-4	295 (54.9)	263 (49.0)	
>=5	179 (33.4)	205 (38.2)	
**Usual bedtime**			
8.00 PM-10.00 PM	71 (13.2)	11 (2.0)	**<0.0001**
10.00 PM-12.00 AM	330 (61.5)	106 (19.7)	
12.00 AM-2.00 AM	119 (22.2)	263 (49.0)	
After 2.00 AM	17 (3.2)	157 (29.2)	
**Usual wake-up time**			
Before 6.00 AM	59 (11.0)	30 (5.6)	**<0.0001**
6.00 AM-8.00 AM	311 (57.9)	53 (9.9)	
8.00 AM-10.00AM	130 (24.2)	225 (41.9)	
10.00AM-12.00 PM	37 (6.9)	229 (42.7)	
**Usual nap length**			
None	267 (49.7)	222 (41.3)	**<0.0001**
Less than 30min	133 (24.8)	90 (16.8)	
30min-1h	106 (19.7)	143 (26.6)	
1h-2h	31 (5.7)	82 (15.2)	
**Daily physical activity**			
None	186 (34.6)	315 (58.7)	**<0.0001**
Less than 30min	134 (25.0)	132 (24.6)	
30min-1h	155 (28.9)	77 (14.3)	
1h-1h30min	47 (8.8)	8 (1.5)	
1h30-2h	15 (2.8)	5 (1.0)	
**Daily hygiene**			
Less than 30min	178 (33.1)	162 (30.2)	**0.009**
30min-1h	240 (44.7)	227 (42.3)	
1h-1h30min	74 (13.8)	102 (19.0)	
1h30min-2h	45 (8.4)	46 (8.6)	
**Daily household activities**			
None	106 (19.7)	69 (12.8)	**<0.0001**
Less than 1h	199 (37.1)	141 (26.3)	
1h-3h	201 (37.4)	234 (43.6)	
3h-6h	31 (5.8)	93 (17.3)	
**Remote work**			
No	452 (84.2)	261 (48.6)	**<0.0001**
Yes	85 (15.8)	276 (51.4)	
**Daily E-working time**			
None	348 (64.8)	228 (42.5)	**<0.0001**
Less than 2h	88 (16.4)	106 (19.7)	
2h-4h	30 (5.6)	96 (17.9)	
4h-6h	28 (5.2)	63 (11.7)	
6h-8h	24 (4.5)	22 (4.1)	
>8h	19 (3.5)	22 (4.1)	
**Daily information tracking time**			
None	100 (18.6)	33 (6.1)	**<0.0001**
Less than 30min	245 (45.6)	146 (27.2)	
30min-1h	137 (25.5)	183 (34.1)	
1h-2h	34 (6.3)	74 (13.8)	
2h-3h	15 (2.8)	43 (8.0)	
>3h	6 (1.1)	58 (10.8)	
**Daily phone calls and SMS**			
Less than 30min	169 (31.5)	105 (19.6)	**<0.0001**
30min-1h	183 (34.1)	117 (21.8)	
1h-2h	110 (20.5)	148 (27.6)	
2h-3h	43 (8.0)	81 (15.1)	
>3h	32 (6.0)	86 (16.0)	
**Daily activities time with family members**			
Less than 1h	213 (39.7)	96 (17.9)	**<0.0001**
1h-2h	189 (35.2)	123 (22.9)	
2h-4h	108 (20.1)	174 (32.4)	
4h-6h	27 (5.0)	144 (26.8)	
**Life satisfaction**			
Not satisfied	38 (7.1)	132 (24.6)	**<0.0001**
Somewhat satisfied	63 (11.7)	125 (23.3)	
Moderately satisfied	181 (33.7)	160 (29.8)	
Very satisfied	182 (33.9)	77 (14.3)	
Extremely satisfied	73 (13.6)	43 (8.0)	

⁎ Stuart-Maxwell test for the marginal homogeneity for two dependent samples.

## 2 Experimental Design, Materials and Methods

### 2.1 Data collection

Data during the home confinement were collected from May 9, to May 30, 2020. Participants were asked to complete in Moroccan Arabic dialect an anonymous online questionnaire consisted of EQ-5D-5L, socio-demographic variables (age, gender, marital status, number of children, educational level, socioeconomic level) and 24 questions corresponding to daily behaviors related to lifestyle (Table 1S, Supplementary material). The online questionnaire was designed following Helsinki's Declaration of ethics. On the main page, a summary of the purpose of the data collection and a letter of consent were presented to the respondents. Access to the questionnaire was only given if the respondent consented to participate. Participants were females and males aged over 18 years.

### 2.2 Health-related quality of life

The health-related quality of life was assessed using the EQ-5D-5L questionnaire [7]. The instrument consists of a descriptive system and a Visual Analog Scale (VAS). The later one allows the individual to appreciate his/her current health states (scale 0–100, where 0=the worse imaginable and 100=the best imaginable). The descriptive system comprises five health dimensions (5D: mobility, self-care, usual activities, pain/discomfort and anxiety/depression), where 5 levels (5L) are used to represent the degree of the health state severity: no problems (level 1), slight problems (level 2), moderate problems (level 3), severe problems (level 4) or extreme problems (level 5). The participant's response was converted into a five-digit number describing the health state, i.e, 51243 is the health state equivalent to extreme problems in mobility, no problems in self-care, slight problems in usual activities, severe problems in pain/discomfort, and moderate problems in anxiety depression. By the use of an appropriate algorithm, the five-digit health states are converted into utility scores, which are available for several countries. When utility scores are missing, it would be acceptable to apply another country's value set to estimate utilities. As the Moroccan value set for the EQ-5D-5L have not yet been developed, we used the France value set to calculate utility scores using EuroQol program [9].

### 2.3 Statistical analysis

The comparison between two independent EQ-5D-5L data samples (example: before vs during the home confinement, healthy vs ill, female vs male) was conducted basing on (1) the global information contained in the utility and VAS scores and (2) the information from each EQ-5D-5L dimension. For utility and VAS which are continuous variables the Mann-Whitney and Kruskal-Wallis tests were used for the global health status pair and multiple comparisons, respectively. For each EQ-5D-5L dimension which characterized by ordinal variable representing the severity level we used the Improved RIDIT approach [8]. This approach takes into account the severity level of the EQ-5D-5L dimensions, which permits to estimate the Absolute Risk Reduction/Absolute Risk Increase (ARR/ARI) and the ordinal odds ratio. The improved RIDIT permits analyzing of the five dimensions of the EQ-5D-5L separately, which gives more precision in understanding the effect of a circumstantial condition (pathology, treatment or experimental condition) on the health status. Herein, we evaluated the effect of the circumstantial home confinement due to the COVID-19 crisis on the health-related quality of life of Moroccan population. Therefore, for each EQ-5D-5L health dimension the HRQoL difference between two independent samples was estimated by the difference between two probabilities (π^+^ and π^−^). π^+^ (π^−^) is the probability that a randomly selected participant from one sample (example, during confinement) is in severe (better) health state than a randomly selected participant from the other sample (example, before confinement). So, if π^+^ - π^−^ is a positive value then the confinement would impact negatively the EQ-5D-5L health dimension, otherwise participants would be in a better health dimension. For example, if π^+^ - π^−^> 0 (if π^+^ - π^−^ < 0) for the pain/discomfort dimension, one says that the confinement increases (decreases) the pain/discomfort by about ARI = π^+^ - π^−^ (ARR = π^−^ - π^+^) percent.

Analysis of the behavior changes due to the home confinement was conducted using the Stuart-Maxwell test for the marginal homogeneity for two dependent samples.

## Ethics Statement

The data collection was designed following Helsinki's Declaration of ethics. The online questionnaire was anonymous and the data were coded. On the main page, a summary of the purpose of the data collection and aa online letter of consent were presented to the respondents. Access to the questionnaire was only given if the respondent consented to participate.

## CRediT authorship contribution statement

**Asmaa Azizi:** Conceptualization, Visualization, Data curation, Writing - original draft, Writing - review & editing. **Doha Achak:** Conceptualization, Visualization, Data curation, Writing - original draft, Writing - review & editing. **Khalid Aboudi:** Formal analysis, Data curation, Writing - review & editing. **Elmadani Saad:** Formal analysis, Writing - review & editing. **Chakib Nejjari:** Formal analysis, Writing - review & editing. **Youness Nouira:** Formal analysis, Data curation, Writing - review & editing. **Abderraouf Hilali:** Conceptualization, Supervision, Writing - review & editing. **Ibtissam Youlyouz-Marfak:** Conceptualization, Supervision, Visualization, Writing - original draft, Writing - review & editing. **Abdelghafour Marfak:** Supervision, Data curation, Methodology, Visualization, Writing - original draft, Writing - review & editing.

## Declaration of Competing Interest

The authors declare that they have no known competing financial interests or personal relationships which have, or could be perceived to have, influenced the work reported in this article.

## Appendix Supplementary materials

Image, application 1

Image, application 2

Image, application 3

Image, application 4

Image, application 5

Image, application 6
