# Supplementary material for: Health-related quality of life and behavior-related lifestyle changes due to the COVID-19 home confinement: Dataset from a Moroccan sample
Source: Data Brief. 2020 Aug 27;32:106239. doi: 10.1016/j.dib.2020.106239 (PMC7449885; doi:10.1016/j.dib.2020.106239)
Supplement: Supplementary file 1 [file mmc1.docx]

**Table 1S**

The Questionnaire used during the home confinement to collect data for health-related quality of life and behaviors.

| 1. **Sociodemographic characteristics** | | | |
| --- | --- | --- | --- |
| Sexe | Female  Male | | |
| Age |  | | |
| Marital status | Single  Married  Separated  Widowed | | |
| Number of children | 0  1-4  >4 | | |
| Educational status | Illiterate  Primary education  Secondary education  University education | | |
| Profession | Student  Worker  No occupation | | |
| Socio economic level | Low  Medium  High | | |
| Presence of disease | No  Yes | | |
| 1. **Health-related quality of life** | | | |
| - 1. **By** placing a tick in one box in each health dimension below (**Mobility, Self-care, Usual activities, Pain/Discomfort and Anxiety/Depression**), please indicate which statements best describe your own health state today. | | | |
| **Mobility** | I have **no problems** in walking about  I have **slight problems** in walking about  I have **moderate problems** in walking about  I have **severe problems** in walking about  **I am unable** to walk about | | |
| **Self-care** | I have **no problems** washing or dressing myself  I have **slight problems** washing or dressing myself  I have **moderate problems** washing or dressing myself  I have **severe problems** washing or dressing myself  **I am unable** to wash or dress my self | | |
| **Usual activities** *(e.g. work, study, housework, family or leisure activities)* | I have **no problems** doing my usual activities  I have **slight problems** doing my usual activities  I have **moderate problems** doing my usual activities  I have **severe problems** doing my usual activities  **I am unable** to do my usual activities | | |
| **Pain/Discomfort** | I have **no** pain or discomfort  I have **slight** pain or discomfort  I have **moderate** pain or discomfort  I have **severe** pain or discomfort  I have **extreme** pain or discomfort | | |
| **Anxiety/Depression** | I am **not** anxious or depressed  I am **slightly** anxious or depressed  I am **moderately** anxious or depressed  I am **severely** anxious or depressed  I am **extremely** anxious or depressed | | |
| - 1. We would like to know how good or bad your health is **TODAY**. This scale is numbered from **0** to **100** (**100** means the best health you can imagine and **0** means the worst health you can imagine). Please enter a value between 0 and 100 to indicate how your health is **TODAY**. | | | |
|   Your health **TODAY** | | | |
| 1. **Behaviors and lifestyle** | | | |
|  |  | **Before confinement** | **During confinement** |
| How many meals do you eat per day? | 1-2  3-4  >=5 |  |  |
| How many times do you snack per day? | None  1-2  3-4  >=5 |  |  |
| What is the interval between meals? | 1-2h  3-4h  >=5h |  |  |
| What is your usual bedtime? | 8.00 PM-10.00 PM  10.00 PM-12.00 AM  12.00 AM-2.00 AM  After 2.00 AM |  |  |
| What is your usual wake-up time? | Before 6.00 AM  6.00 AM-8.00 AM  8.00 AM-10.00 AM  10.00 AM-12.00 PM |  |  |
| How much time per day do you spend napping? | None  Less than 30 min  30min-1h  1h-2h |  |  |
| How much time per day do you devote to physical exercise (sport)? | None  Less than 30 min  30min-1h  1h-1h30min  1h30min-2h |  |  |
| How much time per day do you spend on your daily hygiene? | Less than 30 min  30min-1h  1h-1h30min  1h30min-2h |  |  |
| How much time per day do you spend on household activities? | None  Less than 1h  1h-3h  3h-6h |  |  |
| In your profession, are you required to work remotely? | Yes  No |  |  |
| How much time per day do you devote on E-working? | None  Less than 2h  2h-4h  4h-6h  6h-8h  >8h |  |  |
| How much time per day do you spend on tracking information? | None  Less than 30 min  30min-1h  1h-2h  2h-3h  >3h |  |  |
| How much time per day do you spend on phone calls and sms? | Less than 30 min  30min-1h  1h-2h  2h-3h  >3h |  |  |
| How much time per day do you spend on activities (meals, discussions, board games, console, movies,...) with your family members living with you? | Less than 1h  1h-2h  2h-4h  4h-6h |  |  |
| Are you satisfied with your life? | Not satisfied  Somewhat satisfied  Moderately satisfied  Very satisfied  Extremely satisfied |  |  |
| **During the home confinement, how much do you miss the following activities?** | | | |
| Go to the supermarket | I miss it extremely  I miss it very much  I miss it moderately  I miss it very little  I don’t miss it  Not concerned |  | |
| Go to the mosque | I miss it extremely  I miss it very much  I miss it moderately  I miss it very little  I don’t miss it  Not concerned |  | |
| Go to the cafe | I miss it extremely  I miss it very much  I miss it moderately  I miss it very little  I don’t miss it  Not concerned |  | |
| Go to the popular bath (Hammam) and SPA | I miss it extremely  I miss it very much  I miss it moderately  I miss it very little  I don’t miss it  Not concerned |  | |
| Go to the sports’ club and leisure areas | I miss it extremely  I miss it very much  I miss it moderately  I miss it very little  I don’t miss it  Not concerned |  | |
| Visiting family | I miss it extremely  I miss it very much  I miss it moderately  I miss it very little  I don’t miss it  Not concerned |  | |
| Visiting friends | I miss it extremely  I miss it very much  I miss it moderately  I miss it very little  I don’t miss it  Not concerned |  | |
| Shopping | I miss it extremely  I miss it very much  I miss it moderately  I miss it very little  I don’t miss it  Not concerned |  | |
| Go to restaurants | I miss it extremely  I miss it very much  I miss it moderately  I miss it very little  I don’t miss it  Not concerned |  | |
