# Supplementary material for: Health-related quality of life and behavior-related lifestyle changes due to the COVID-19 home confinement: Dataset from a Moroccan sample
Source: Data Brief. 2020 Aug 27;32:106239. doi: 10.1016/j.dib.2020.106239 (PMC7449885; doi:10.1016/j.dib.2020.106239)
Supplement: Supplementary file 2 [file mmc2.docx]

**Table 2S**

| **Variables** | **MO** | | **SC** | | **UA** | | **PD** | | **AD** | |
| --- | --- | --- | --- | --- | --- | --- | --- | --- | --- | --- |
|  | **ARI**  **[95% CI]** | **Odds**  **[95% CI]** | **ARI**  **[95% CI]** | **Odds**  **[95% CI]** | **ARI**  **[95% CI]** | **Odds**  **[95% CI]** | **ARI**  **[95% CI]** | **Odds**  **[95% CI]** | **ARI**  **[95% CI]** | **Odds**  **[95% CI]** |
| **Sexe**  Male (ref)  Female | **0.07**  **[0.01 ; 0.13]** | **1.94**  **[1.38 ; 2.71]** | 0.005  [-0.02 ; 0.04] | 1.18  [0.52 ; 2.69] | 0.02  [-0.04 ; 0.09] | 1.17  [0.79 ; 1.73] | **0.2**  **[0.12 ; 0.28]** | **2.65**  **[2.09 ; 3.36]** | **0.14**  **[0.04 ; 0.23]** | **1.54**  **[1.18 ; 2.00]** |
| **Age**  18-30 (ref)  31-50  > 50 | **0.06**  **[0.009 ; 0.12]**  **0.20**  **[0.11 ; 0.29]** | **1.91**  **[1.27 ; 2.86]**  **4.03**  **[2.48 ; 6.54]** | -0.007  [-0.04 ; 0.02]  **0.07**  **[0.01; 0.12]** | 0.75  [0.16 ; 3.67]  **3.60**  **[1.59 ; 8.18]** | 0.03  [-0.04 ; 0.10]  -0.01  [-0.11 ; 0.09] | 1.21  [0.81 ; 1.80]  0.91  [0.42 ; 1.99] | 0.04  [-0.04 ; 0.13]  **0.15**  **[0.03 ; 0.27]** | 1.23  [0.87 ; 1.72]  **1.86**  **[1.20 ; 2.90]** | -0.02  [-0.12 ; 0.07]  -0.21  [-0.35 ; -0.06] | 0.92  [0.61 ; 1.40]  0.51  [0.10 ; 2.60] |
| **Marital status**  Single (ref)  Married  Separated  Widowed | **0.08**  **[0.025 ; 0.14]**  -  - | **2.07**  **[1.46 ; 2.92]**  -  - | 0.02  [-0.02 ; 0.05]  -  - | 1.65  [0.83 ; 3.29]  -  - | -0.01  [-0.08 ; 0.05]  -  - | 0.91  [0.55 ; 1.51]  -  - | **0.09**  **[0.01 ; 0.17]**  -  - | **1.50**  **[1.12 ; 2.01]**  -  - | -0.12  [-0.21 ; 0.02]  -  - | 0.69  [0.38 ; 1.26]  -  - |
| Profession  Students (ref)  Workers  No occupation | 0.08  [0.01 ; 0.15]  0.18  [0.09 ; 0.27] | 2.88  [1.98 ; 4.18]  5.83  [3.61 ; 9.41] | 0.02  [-0.02 ; 0.05]  0.03  [-0.02 ; 0.07] | 1.88  [0.92 ; 3.85]  2.37  [0.84 ; 6.71] | -0.007  [-0.09 ; 0.07]  0.06  [-0.06 ; 0.17] | 0.95  [0.51 ; 1.78]  1.40  [0.82 ; 2.36] | 0.04  [-0.06 ; 0.14]  **0.16**  **[0.03 ; 0.29]** | 1.19  [0.79 ; 1.79]  **1.95**  **[1.29 ; 2.96]** | -0.07  [-0.19 ; 0.04]  0.05  [-0.1 ; 0.21] | 0.79  [0.42 ; 1.47]  1.17  [0.70 ; 1.97] |
| **Presence of disease**  No (ref)  Yes | **0.11**  **[0.05 ; 0.19]** | **2.36**  **[1.54 ; 3.64]** | **0.04**  **[0.0007;0.07]** | **2.58**  **[1.21 ; 5.54]** | **0.08**  **[0.0004;0.16]** | **1.60**  **[1.06 ; 2.42]** | **0.24**  **[0.15 ; 0.34]** | **2.62**  **[1.92 ; 3.58]** | 0.07  [-0.04 ; 0.19] | 1.25  [0.84 ; 1.86] |

Association between the EQ-5D-5L health dimensions and the socio-demographic characteristics of individuals during the home confinement.

MO: Mobility, SC: Self-care, UA: Usual activities, PD: Pain/Discomfort, AD: Anxiety/Depression, ARI: Absolute Risk Increase and CI: Confidence Interval.
