# Supplementary material for: Health-related quality of life and behavior-related lifestyle changes due to the COVID-19 home confinement: Dataset from a Moroccan sample
Source: Data Brief. 2020 Aug 27;32:106239. doi: 10.1016/j.dib.2020.106239 (PMC7449885; doi:10.1016/j.dib.2020.106239)
Supplement: Supplementary file 3 [file mmc3.docx]

**Table 3S**

The profile of missing activities during the home confinement.

| **How much do you miss the following activities?** | **During confinement**  **n (%)** |
| --- | --- |
| **Go to the supermarket** |  |
| I miss it extremely | 9 (1.7) |
| I miss it very much | 37 (6.9) |
| I miss it moderately | 57 (10.6) |
| I miss it very little | 171 (31.8) |
| I don’t miss it | 237 (44.1) |
| Not concerned | 26 (4.8) |
| **Go to the mosque** |  |
| I miss it extremely | 113 (21.0) |
| I miss it very much | 87 (16.2) |
| I miss it moderately | 43 (8.0) |
| I miss it very little | 82 (15.3) |
| I don’t miss it | 163 (30.4) |
| Not concerned | 49 (9.1) |
| **Go to the cafe** |  |
| I miss it extremely | 31 (5.8) |
| I miss it very much | 58 (10.8) |
| I miss it moderately | 63 (11.7) |
| I miss it very little | 72 (13.4) |
| I don’t miss it | 250 (46.6) |
| Not concerned | 63 (11.7) |
| **Go to the popular bath (Hammam) and SPA** | |
| I miss it extremely | 84 (15.6) |
| I miss it very much | 99 (18.4) |
| I miss it moderately | 85 (15.8) |
| I miss it very little | 94 (17.5) |
| I don’t miss it | 145 (27.0) |
| Not concerned | 30 (5.6) |
| **Go to the sports’ club and leisure areas** | |
| I miss it extremely | 74 (13.8) |
| I miss it very much | 92 (17.1) |
| I miss it moderately | 61 (11.4) |
| I miss it very little | 91 (16.9) |
| I don’t miss it | 180 (33.5) |
| Not concerned | 39 (7.3) |
| **Visiting family** |  |
| I miss it extremely | 139 (25.9) |
| I miss it very much | 113 (21.0) |
| I miss it moderately | 75 (14.0) |
| I miss it very little | 98 (18.2) |
| I don’t miss it | 85 (15.8) |
| Not concerned | 27 (5.0) |
| **Visiting friends** |  |
| I miss it extremely | 109 (20.3) |
| I miss it very much | 115 (21.4) |
| I miss it moderately | 81 (15.1) |
| I miss it very little | 101 (18.8) |
| I don’t miss it | 111 (20.7) |
| Not concerned | 20 (3.7) |
| **Shopping** |  |
| I miss it extremely | 61 (11.4) |
| I miss it very much | 84 (15.6) |
| I miss it moderately | 81 (15.1) |
| I miss it very little | 130 (24.2) |
| I don’t miss it | 159 (29.6) |
| Not concerned | 22 (4.1) |
| **Go to restaurants** |  |
| I miss it extremely | 66 (12.3) |
| I miss it very much | 74 (13.8) |
| I miss it moderately | 82 (15.3) |
| I miss it very little | 107 (19.9) |
| I don’t miss it | 164 (30.5) |
| Not concerned | 44 (8.2) |
